# CHILDHOOD SOCIOECONOMIC POSITIONING AND LATE-LIFE COGNITIVE FUNCTIONING: A CRITICAL REVIEW

**DOI:** 10.1093/geroni/igac059.2474

**Published:** 2022-12-20

**Authors:** Hanamori Skoblow, Christine Proulx

**Affiliations:** University of Missouri, Columbia, Missouri, United States; University of Missouri, Columbia, Missouri, United States

## Abstract

The predictors of cognitive functioning are varied and complex. Gerontologists are increasingly interested in the long arm of childhood, suggesting that late-life cognitive functioning may be partly the result of influences across the life span. Research shows that childhood economic hardship is associated with disparities in cognitive functioning in older adulthood. Framed by the life course perspective, we reviewed 27 articles that examine associations between childhood socioeconomic positioning (SEP), commonly assessed via parents’ educational attainment, and late-life cognitive functioning in 11 different US datasets. The influence of childhood SEP on cognitive functioning is stronger when cognitive functioning is assessed at a single time point rather than as change over time, suggesting that childhood SEP might not affect the rate at which cognition declines in later life, but does impact where decline begins. The majority of research supported the pathway hypothesis, suggesting that childhood SEP’s influence on adult cognitive functioning works primarily through the mechanism of adults’ own educational attainment and SEP. Several studies support the accumulation of (dis)advantage hypothesis and point to the compensatory potential of upwards social mobility. Support for the latency model, which posits that early-life economic hardship results in enduring outcomes that influence cognitive functioning in older adulthood, above and beyond one’s adult SEP, is present, although weaker than the other hypotheses. Implications include strengthening policies that relieve economic strain and promote educational access among families with young children and also among young adults, as addressing the precursors of cognitive functioning in tomorrow’s older adults is paramount.

